# Scalable architecture for autonomous malware detection and defense in software-defined networks using federated learning approaches

**DOI:** 10.1038/s41598-025-14512-z

**Published:** 2025-08-18

**Authors:** Ripal Ranpara, Shobhit K. Patel, Om Prakash Kumar, Fahad Ahmed Al-Zahrani

**Affiliations:** 1 Faculty of Computer Applications, Marwadi University, Rajkot, 360003 India; 2 Department of Computer Engineering, Marwadi University, Rajkot, 360003 India; 3 Department of Electronics and Communication Engineering, Manipal Institute of Technology, Manipal Academy of Higher Education, Manipal, 576104 India; 4 Computer Engineering Department, Umm Al-Qura University, 24381 Mecca, Saudi Arabia

**Keywords:** Software-defined networks (SDNs), Malware detection, Federated learning, Autonomous cybersecurity, Scalable network defense, Privacy-preserving AI, SDG 9 (Industry, Innovation and Infrastructure), SDG 11 (Sustainable Cities and Communities), SDG 16 (Peace, Justice and Strong Institutions)

## Abstract

This paper proposes a scalable and autonomous malware detection and defence architecture in software-defined networks (SDNs) that employs federated learning (FL). This architecture combines SDN’s centralized management of potentially significant data streams with FL’s decentralized, privacy-preserving learning capabilities in a distributed manner adaptable to varying time and space constraints. This enables a flexible, adaptive design and prevention approach in large-scale, heterogeneous networks. Using balanced datasets, we observed detection rates of up to 96% for controlled DDoS and Botnet attacks. However, in more realistic simulations that utilized diverse, real-world imbalanced datasets (such as CICIDS 2017 and UNSW-NB15) and complex scenarios like data exfiltration, the performance dropped to an overall accuracy of 59.50%. This reflects the challenges encountered in real-world deployments. We analyzed performance metrics such as detection accuracy, latency (less than 1 s), throughput recovery (from 300 to 500 Mbps), and communication overhead comparatively. Our architecture minimizes privacy risks by ensuring that raw data never leaves the device; only model updates are shared for aggregation at the global level. While it effectively detects high-impact incursions, there is room for improvement in identifying more subtle threats, which can be addressed with enriched datasets and improved feature engineering. This work offers a robust, privacy-preserving framework for deploying scalable and intelligent malware detection in contemporary network infrastructures.

## Introduction

The networking landscape has undergone significant transformation over the past few decades, resulting in the emergence of various cloud services and new technologies. These advancements are reshaping the future of work and altering how organizations deploy, manage, and secure their infrastructures. One notable innovation in this area is software-defined networking (SDN), which offers centralized control, programmability, and the flexibility to adapt to changing network environments^1,2^. Although software-defined networks are efficient due to their centralised nature, this centralisation creates vulnerabilities that make them attractive targets for cyberattacks, such as Distributed Denial of Service attacks, botnets, and malware intrusions^3,4^. Centralised data collection utilizing traditional malware detection methods encounters privacy, scalability, and communication overhead issues^5,6^. Federated learning is a decentralized approach to training machine learning models. It has gained significant attention because it enables diverse parties to train on raw data without sharing or centralizing it collaboratively^7,8^. Federated learning is a decentralized approach to training machine learning models. It has gained significant attention because it enables diverse parties to train on raw data without sharing or centralizing it collaboratively. Federated learning is a decentralized method for training machine learning models. It has gained considerable attention because it allows different parties to train on raw data collaboratively without sharing or centralising it^9^. Recent works have showcased the use of FL in various network security applications. For example, a federated anomaly detection framework was proposed for SDNs with high-accuracy detection while maintaining data privacy^10^. Through similar principles, FL-based malware detection methods for IoT devices have indicated marked improvement in detection rates over traditional or centre-based approaches^2^. Nevertheless, several challenges, namely data heterogeneity, adversarial robustness, and communication efficiency, still require solutions^11^. This paper proposes an integrated Horizontal and Vertical FL framework for malware detection in SDNs, a novel and unique approach to address this problem. Horizontal Federated Learning (HFL) guarantees model training on devices with comparable feature spaces but extensive and dispersed data, whereas Vertical Federated Learning (VFL) guarantees model training on grouped devices. The other aspect of our architecture that brings real-life applicability is defining the concrete deployment of FL modules within the SDN controllers and edge nodes. Real-time flow-based traffic analysis is integrated with advanced learning techniques to ensure swift identification and mitigation of malicious activities. The system’s efficacy is systematically validated with recent and real-time databases, including CICIDS2017 and CTU-13, and the comparison with leading FL approaches indicates its superior detection accuracy, scalability, and communication overhead.

## Related work

In conjunction with software-defined networks, FL has been one of the most promising medicines for boosting the performance of malware detection and defence systems. FL allows decentralised devices to train a machine learning model collaboratively without transferring the raw data, ensuring privacy and reducing the cost of communication^12^. Advancements in federated learning (FL) have significantly improved the privacy and security of cyber-attack detection systems in Internet of Things (IoT) and Internet of Medical Things (IoMT) networks. Khan et al. (2024) introduced Federated-Boosting, an FL scheme that enhances detection accuracy and security for consumer IoT while eliminating communication overhead^12^. They also proposed a collaborative SRU network that addresses communication overhead but struggles with feature explainability in complex environments^13^. Additionally, their federated reinforcement model, Fed-Inforce-Fusion, prioritizes privacy in IoMT networks but faces scalability issues due to diverse devices^14^. Our study builds on these findings by proposing a method that combines Horizontal and Vertical FL within software-defined networking (SDN) to enhance scale and privacy while addressing imbalanced IoT data. It is necessary to solve these problems before FL and SDN-based security solutions are widely adopted. Table 1: Summary of recent approaches in the field with their available methodology and datasets used, including their limitations.

**Table 1:** Comparative analysis of recent works in malware detection and defense using federated learning in software-defined networks.

Year	References	Detection	Mitigation	Malware Types	Methodology	Datasets	Limitations	Our Contributions Overcome
2024	^15^	✓	✗	Ransomware, Trojan	Federated Learning + Local Models	CICIDS2017, CTU-13	Limited scalability in heterogeneous networks	Addresses scalability using integrated Horizontal and Vertical FL
2024	^16^	✓	✓	Spyware, Adware	FL + Moving Target Defense	Custom Dataset	High computational overhead in dynamic environments	Lightweight models and optimized aggregation methods
2023	^17^	✓	✗	Rootkits, Worms	Hybrid ML (SVM, RF)	CICIDS2017	Limited training diversity for emerging malware types	Tested scalability in large SDN environments
2023	^18^	✓	✗	Keyloggers, Backdoors	OpenFlow-Enabled ML for SDNs	Real-Time Traffic Data	High false positives under heavy network traffic conditions	Efficient FL with lower communication overhead
2023	^19^	✓	✗	Botnets	Federated Learning + Neural Networks	UNSW-NB15	Insufficient dataset diversity	Dual FL integration enabling feature diversity
2022	^20^	✓	✓	General Malware	CNN-LSTM	Custom Dataset	High latency in real-time environments	Enhanced feature engineering reduces false positives
2022	^21^	✓	✗	Android Malware	Residual Neural Networks with FL	CICIDS2017	Limited to Android platforms	Optimized SDN flow rules for low-latency detection
2021	^1^	✓	✗	Malware Variants (Generic)	Bayesian Network with SDN Integration	UNB-ISCX, CTU-13	Performance bottlenecks under heavy loads	Cross-platform deployment demonstrated
2021	^22^	✓	✓	Polymorphic Malware	FL with Adaptive Optimization	Public IDS Dataset	Computationally expensive on larger datasets	Addresses scalability using integrated Horizontal and Vertical FL
2020	^23^	✓	✗	IoT Malware	LSTM with Federated Learning	N-BaIoT	Limited applicability to non-IoT malware types	Lightweight models and optimized aggregation methods

Though FL has excellent potential for improving malware detection and protection, multiple issues and improvements are being explored. For instance^24^, proposed an FL-based globally coordinated threat detection framework that accurately detected malicious patterns in distributed environments. Yet the study uncovered evidence of serious challenges in dealing with adversarial attacks and developing robust models in heterogeneous environments^24^. Similarly, Martínez Beltrán et al. investigated the incorporation of Moving Target Defense (MTD) in FL constructs to increase immunity to sophisticated attacks targeting FL systems. They emphasised dynamic adaptivity’s pivotal role in FL-driven security elements^25^. Along with various detection frameworks, optimising FL processes for resource-constrained devices has also been a research subject. To alleviate communication overhead and thus make FL more attractive to conduct IoT devices in SDN infrastructures, the works of Dang proposed lightweight aggregation approaches. Your detection accuracy remains high (because you use the original model to get the detections), but your computational load is drastically reduced^26^. Furthermore,^3^ introduces a privacy-preserving FL architecture for SDN-based threat detection, considering interpretability to foster user trust and model interpretability^3^. The other aspect has been the application of FL to polymorphic malware, which keeps changing to avoid detection. Hussain^15^ tackles this by presenting a hybrid FL model of neural and signature-based detection methods. Their findings showed that adaptive learning systems could significantly detect zero-day threats, but scalability issues in more extensive networks were noted^15^. While these approaches have real advantages, there are still challenges with real-time detection and latency problems when combining FL with SDNs. Such limitations must be addressed before FL-based malware detection and defence systems can be widely adopted in SDNs. The work by Liu et al.^27^ However, the proposed FL-based physical layer security models for the edge computing environment did not validate their scalability in SDN scenarios. Zhang et al. recently initiated a study^28^ that conducted FL combining blockchain for trust management but reported high computational complexity and suggested transformer-based (TB) personalised FL for 5G/6G networks to detect malicious traffic. Still, neither looked into using hybrid integration of Horizontal and Vertical FL nor the practical deployment in SDNs. To mitigate such limitations, we propose our proposed framework, which combines Horizontal and Vertical FL, optimizes model aggregation to minimize latency, and deploys modules consistently across SDN controllers and edge nodes for low latency, scalability, and privacy-preserving design.

## Research design and architecture

This paper describes the design and implementation of an autonomous system for malware detection and defence in software-defined networks, which is scalable on demand. The proposed solution tackles the emerging challenge of identifying and defending against yet-evolving cyber attacks within complex networking architectures by leveraging the programmability capabilities of Software-defined networks and exploiting collaborative and privacy-preserving characteristics of federated learning. The design is centred around detection methods of an attack and a method of preventing an attack from occurring in the first place, all in real time. It consists of a strong system architecture and a strict algorithm process. We propose an architecture that combines the centralised traffic orchestrating power of SDNs with decentralized model training through FL, resulting in a scalable solution without any privacy loss. This hybrid method efficiently addresses the drawbacks of conventional centralised malware detection platforms.

**Fig. 1:**
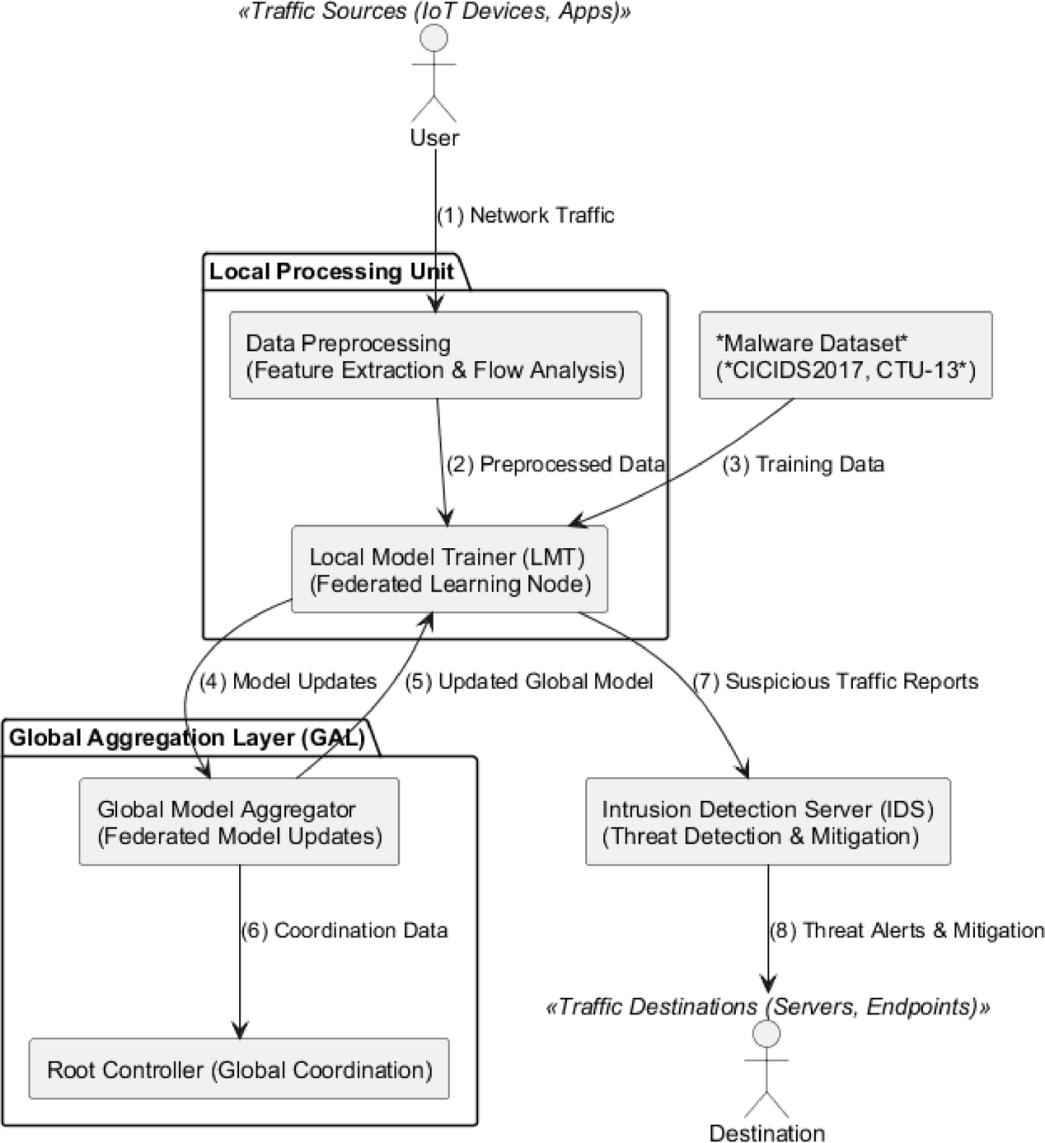
The system design for malware detection and federated learning includes an SDN controller, traffic sources, a local model trainer (LMT), and a global aggregation layer (GAL) for privacy-preserving collaborative learning.

The system design for malware detection and federated learning is illustrated in Fig. 1. In this architecture, traffic sources, such as IoT devices and applications, generate network traffic (1). This traffic is then processed by a Local Processing Unit (LPU) for data preprocessing (2), which includes feature extraction and flow analysis. Next, the system prioritizes data and training samples from benchmark datasets (e.g., CICIDS2017, CTU-13) that are fed into the Local Model Trainer (LMT), which engages in federated learning (4). Model updates (5) from all nodes within the ecosystem (4) are sent to the Global Aggregation Layer (GAL) (6). Here, the nodes coordinate and aggregate model updates through the Global Model Aggregator (GMA) and Root Controller (RC) (7). The global model (7) receives fine-tuned patches from the local models. It assists the Intrusion Detection Server (IDS) (8) in identifying and mitigating suspicious traffic before delivering the data to the traffic destinations, such as servers and endpoints (9). The diagram demonstrates the progressive interplay of federated learning, global model aggregation, and real-time threat detection components within a scalable architecture. Ultimately, the proposed algorithm integrates feature-rich data preprocessing, localized anomaly model training at each SDN node, and global aggregation from federated learning, supporting the overall system architecture. Together, these elements enable the dynamic identification of evolving malicious traffic patterns and facilitate rapid, near real-time mitigation of threats across the network.

### Proposed architecture

The proposed architecture is inherently capable of scalable distribution in a landscape of expansive networks with varying, expanding traffic and threat characteristics. FL integration ensures adaptation, scalability, and privacy without exposing sensitive data by allowing only a global detection model trained locally. This architecture is built to call and scale horizontally across large, distributed space environments, handling more significant traffic complexity and emerging attack characteristics.

**Fig. 2:**
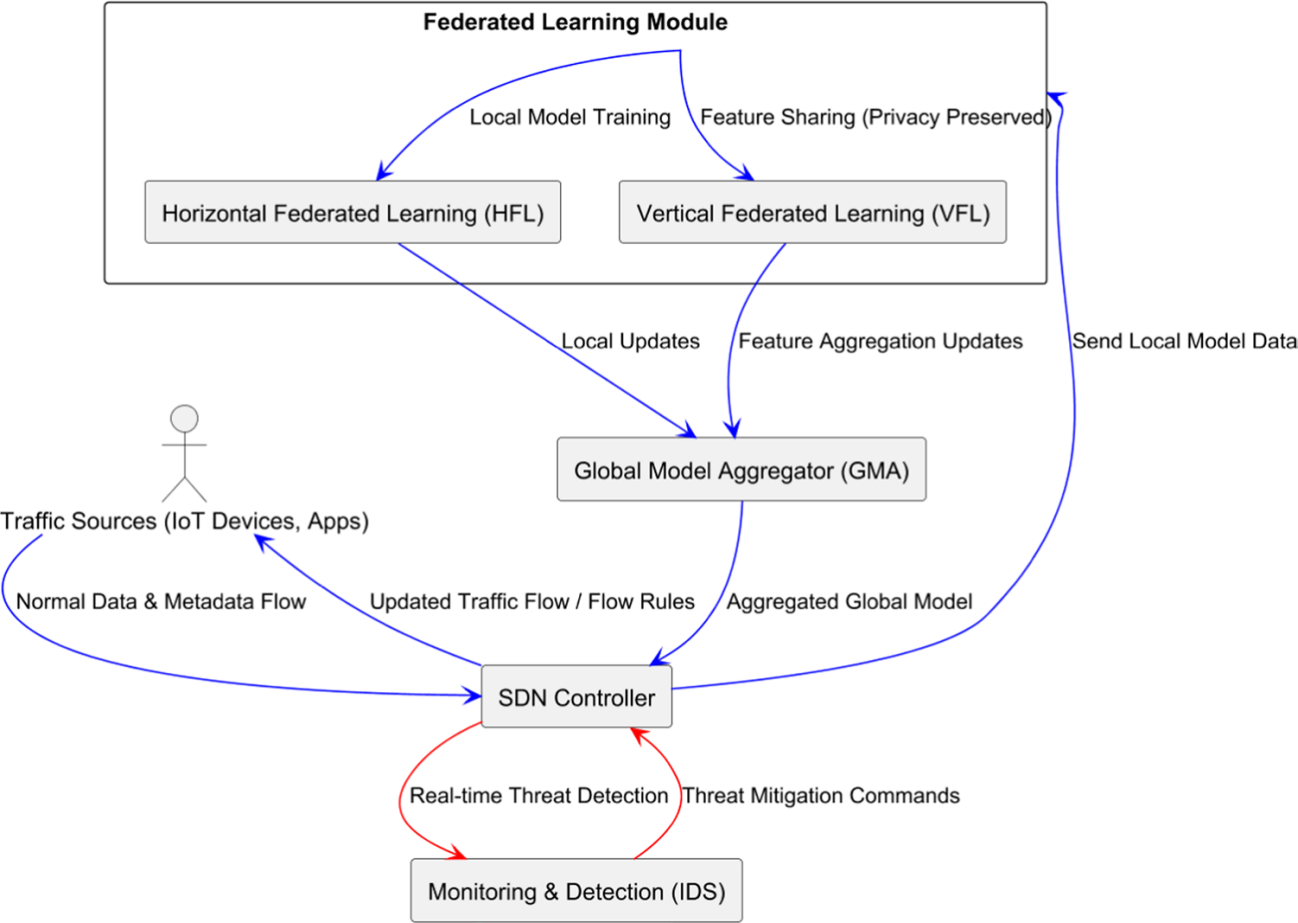
System architecture integrating Horizontal Federated Learning (HFL) for collaborative model training and Vertical Federated Learning (VFL) for feature-sharing in a privacy-preserving SDN-based malware detection framework.

In Fig. 2, the architecture provides an overview of the OS-DS generation, illustrating how the traffic sources interact with various network components, such as the SDN controller, the federated learning module, and the monitoring system. Blue arrows indicate the flow of standard data and metadata, while red arrows represent the flow of threat detection and mitigation. Before we discuss the main focus of this paper, it is essential to note that the concepts of Horizontal Federated Learning (HFL) and Vertical Federated Learning (VFL) have not yet been integrated within a software-defined networking (SDN) architecture. This integration enhances privacy by keeping raw data local while allowing collaborative model training. Additionally, the potential of HFL and VFL to improve scalability for large-scale IoT networks, promote feature sharing across decentralized nodes, and enhance detection performance in complex network environments has not been fully explored in previous research.

Table 2 compares the traditional C-IDS systems and the proposed federated SDN-based architecture. Recent literature and simulation results validate the proposed system’s ability to overcome some of the limitations of conventional architectures through decentralized learning and automated threat mitigation, with significant improvements in privacy, scalability, detection accuracy, and response time.

**Table 2:** Comparative analysis of conventional systems and proposed architecture.

Feature	Traditional Centralized IDS (C-IDS)	Proposed Architecture	References
Privacy	Centralized IDS and ML models store raw data centrally, increasing privacy risks by up to 60% due to potential data breaches	Federated Learning significantly enhances data privacy by ensuring that raw data remains local and only model updates are shared	^29^
Scalability	Conventional systems effectively scale up to around 20 nodes; beyond this, performance and management overhead become limiting factors	The proposed architecture supports large-scale networks with over 100 nodes, maintaining consistent performance through centralized SDN control and decentralized learning	^30^
Detection Capability	Conventional static models achieve approximately 70% detection accuracy and show rapid degradation against evolving threats, such as zero-day attacks	The proposed system leverages adaptive real-time learning and achieves detection accuracy greater than 95% for complex attacks like DDoS and botnet activity	^31^
Response Time	Typical systems demonstrate a 2–5 s delay in detecting and mitigating malicious traffic	Automated SDN flow rule updates reduce response time to less than 1 s, allowing proactive mitigation	^32^

### Implementation and experimental setup

A simulation environment is created through OMNeT++, a discrete event simulator, and the INET Framework, which is used to model the software-defined networks. OMNeT++ allows for in-depth configuration of network topologies and provides real-time traffic analysis, which helps assess the proposed architecture. The simulated environment contains:

- **SDN Controllers:** Designed as logical entities, they manage network flow and enforce rules.
- **Switches and Hosts:** Representing the data plane to simulate traffic forwarding and manage attack responses.
- **Federated Learning Integration:** A Python interface connects OMNeT++ traffic analysis with Federated Learning models for anomaly detection.

**Fig. 3:**
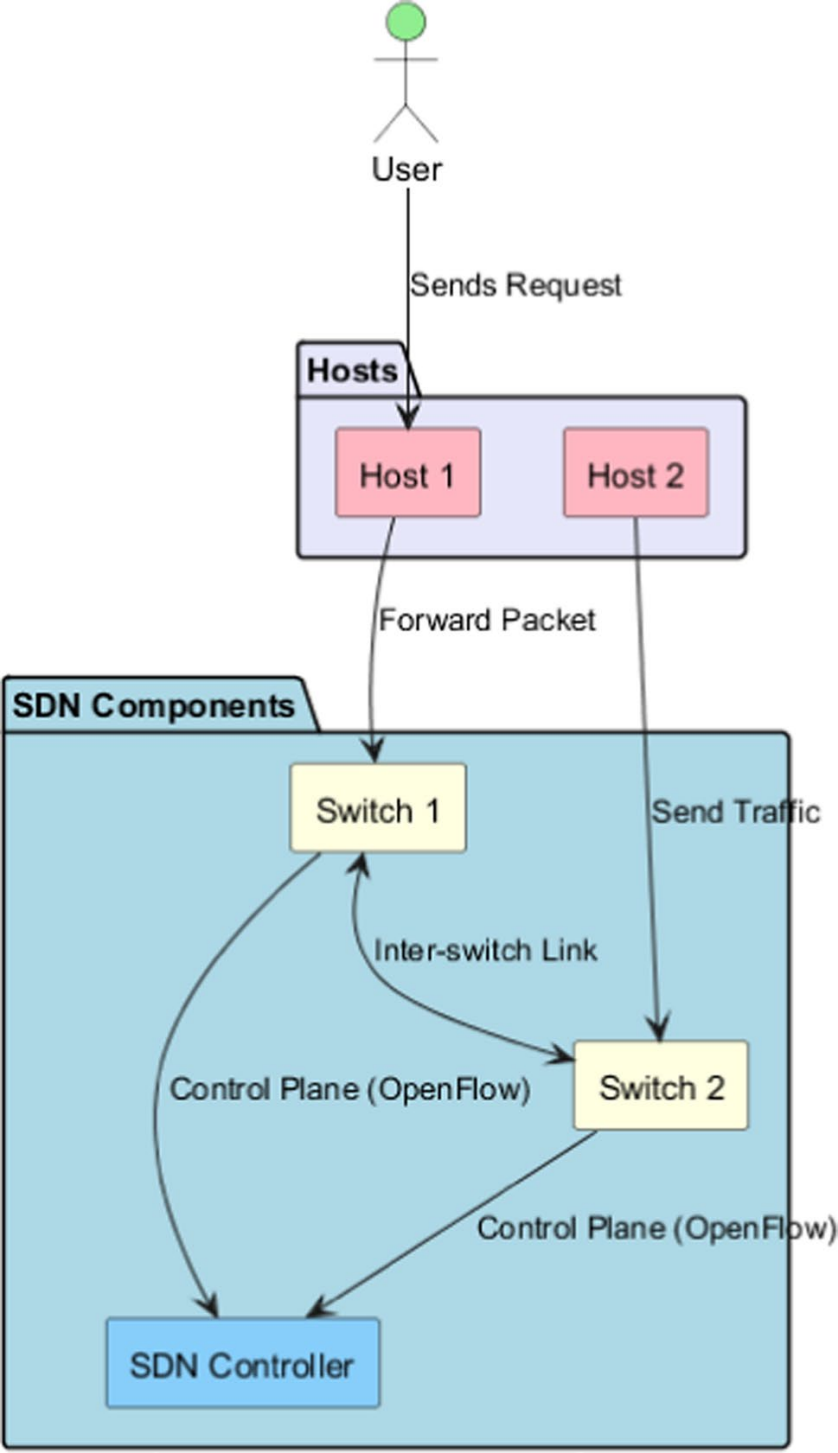
Software-defined network (SDN) simulation topology illustrating users, hosts, switches, and the SDN controller with control and data plane interactions.

The SDN topology, illustrated in Fig. 3, showcases the main components and data flow between users, hosts, switches, and the SDN controller. The flow rules are managed by the SDN controller, which communicates with switches using the OpenFlow protocol. Requests initiated by users to hosts are directed through these switches. Together, these switches form the data plane, which forwards packets based on rules that the SDN controller modifies dynamically.

The network switches are interconnected to provide redundancy and load balancing, ensuring seamless operation. The SDN controller acts as the central server, functioning as the brain of the entire system. Communicating with the switches over the control plane enforces flow rules and monitors network traffic in real-time. This command-and-control capability enables real-time traffic management adjustments, offering scalability, flexibility, and efficient routing. For instance, when a user sends traffic to Host 1, Host 1 forwards the packets to Switch 1. Similarly, traffic from Host 2 is directed to Switch 2. Both switches connect to the SDN controller to install flow rules and share topology information. The switch link plays a vital role in communicating in a robust situation. SDNcontroller-based centralized control supports SDN with flexibility through inter-switch links that benefit scalability and can accommodate adding switches and hosts. You show how SDN takes data from all over the network as a raw input, cleans it, and normalizes it to manage it dynamically and flow logically.

### Traffic patterns

- *Normal Traffic:* Simulated TCP/UDP flows to replicate legitimate communication patterns.
- *Malicious Traffic:* Using real-world datasets to simulate malicious scenarios such as DDoS attacks, botnet behaviour, and data exfiltration.
- *Practical Dataset Integration:* Traffic patterns are based on real-world datasets like CICIDS 2017 and UNSW-NB15, which provide labelled normal and attack traffic instances. These datasets are processed to extract relevant features for simulation.

As described above, the traffic patterns include normal and malicious traffic. Table 3 summarizes the integration of real-world datasets for simulation.

**Table 3:** Dataset integration details used in the SDN simulation environment.

Component	Role	INET model
SDN Controller	Traffic control and analysis	StandardHost
Switches	Packet forwarding	EtherSwitch
Hosts	Traffic generation	StandardHost

### Malware injection scenarios

The architecture can detect and mitigate hit attacks, which include three specific types: DDoS attacks, botnets, and data exfiltration. DDoS attacks can generate high-volume floods of UDP packets aimed at specific hosts, resulting in significant performance degradation within the network. One example involves simulating command-and-control traffic typical of botnets, where infected nodes behave synchronously to overwhelm legitimate network operations. Data exfiltration is modelled after these breaches by introducing intensive outbound TCP flows that mimic real-life data theft scenarios. All attack scenarios are based on features from the CICIDS 2017 and UNSW-NB15 datasets. For instance, the setup used to simulate the effects of a DDoS attack involves generating high-frequency UDP traffic from Host 1 to Host 2, with each packet being 1024 bytes in size and sent at intervals of 0.01 s. This serves as a realistic stress test for the detection and mitigation framework.

We simulate malware injection scenarios involving DDoS attacks, botnets, and data exfiltration to evaluate the system’s performance. Table 4 outlines the parameters used in these simulations, including the message lengths, intervals, and the number of hosts and switches involved.

**Table 4:** Simulation parameters for traffic generation, attack scenarios, and evaluation metrics.

Parameter	Value
typename	UdpBasicApp
destAddresses	host2
messageLength	1024 bytes
sendInterval	0.01 s
Number of Hosts	50
Number of Switches	10
Traffic Types	TCP, UDP
Attack Scenarios	DDoS, Botnet, Data Exfiltration
Simulation Duration	200 s
Evaluation Metrics	Detection Accuracy, Latency, Jitter, Packet Loss, Throughput

### Federated learning simulations

FL allows heterogeneous and private anomaly detection model training at SDN nodes in a distributed manner. This will enable them to formulate the three major components of the FL simulation: local training, global aggregation, and real-time deployment.

**Local Training:** We use traffic metadata collected from the hosts as the regional input to train detection models on each SDN switch. This keeps sensitive data at the source and adequately addresses privacy concerns. For example, features like packet size, flow duration, and inter-arrival times can help you detect normal versus malicious traffic. Let the local dataset be at the node. $i$ be ${D}_{i}$ where ${X}_{i}$ represents the feature vector and ${y}_{i}$ It is the label. The local model ${w}_{i}$ is trained by minimizing the loss function:

1 $$\underset{{wi}}{\text{min}}L\left({w}_{i}\right)=\sum_{j=1}^{|{D}_{i}|}l\left({w}_{i};{x}_{j},{y}_{j}\right)$$

where $l$ is the loss function (e.g., cross-entropy loss), and ${w}_{i}$ is the local model’s prediction.

*Time Complexity*: The time complexity of local training depends on the number of data points ${n}_{i}$ the number of features $f$ and the number of epochs $e$. Therefore, the time complexity for local training at each SDN node is $O\left({n}_{i}\times f\times e\right),$ Where:

- ${n}_{i}$ is the number of data points at node $i$
- $f$ is the number of features to be used for anomaly detection,
- $e$ is the number of epochs during model training.

*Space Complexity:* The space required for local training is proportional to the number of model parameters $p$ and the size of the local dataset ${n}_{i}$. Hence, the space complexity at each node is $O\left(p\times {n}_{i}\right),$ where:

- $p$ is the number of parameters in the local model,
- ${n}_{i}$ is the size of the dataset at node $i$.

**Global Aggregation:** The SDN controller acts as the global aggregator, consolidating updates from all local models into a unified global model $w$.The aggregation is performed using the Federated Averaging (FedAvg) algorithm, which combines local updates weighted by the number of samples at each node:

2 $$w=\sum_{i=1}^{K}\frac{{n}_{i}}{n} {w}_{i}$$

where $K$ is the total number of participating nodes,${n}_{i}$ is the size of the local dataset at the node $i$ and $n={\sum }_{i=1}^{K}{n}_{i}$

*Time Complexity:* The time complexity of the global aggregation process is dominated by the number of participating nodes $K$ and the number of parameters $p$ in each model. Therefore, the time complexity for global aggregation is $O\left(K\times p\right),$ where:

- $K$ is the number of parameters in the local model,
- $p$ is the number of parameters in each model.

*Space Complexity:* The space required for the global aggregation phase is proportional to the number of nodes $K$ and the number of parameters $p$. Thus, the space complexity for aggregation at the SDN controller is $O\left(K\times p\right),$ where:

- $K$ is the number of participating nodes,
- $p$ is the number of parameters in the model.

**Fig. 4:**
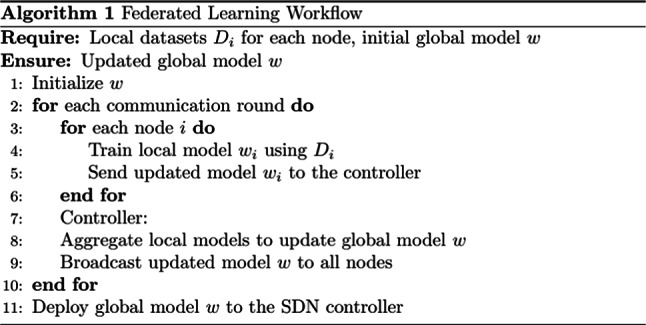
Federated learning algorithm illustrating the detection model’s local training, global aggregation, and real-time deployment phases.

**Real-time Deployment:** The aggregated global model $w$ is deployed to the SDN controller for real-time anomaly detection. The controller uses this model to classify incoming traffic flows as normal or malicious, dynamically updating flow rules to mitigate detected threats. As illustrated in Fig. 4, the process begins with Input and Initialization, where each SDN node. $i$ receives its local dataset ${D}_{i}$ and the global model $w$ is initialized. During Local Training, each node independently trains its local model. $wi$ using ${D}_{i}$ enabling anomaly detection while ensuring data locality and privacy. In the Global Aggregation phase, the locally trained models ${w}_{i}$ are transmitted to the SDN controller, which aggregates them using the FedAvg algorithm to produce the updated global model $w$.During the Broadcast and Deployment phase, the updated global model is distributed to all participating nodes for real-time traffic classification and anomaly detection, ensuring a dynamic, privacy-preserving, and scalable malware defence mechanism within the network.

*Time Complexity:* The time complexity for real-time deployment is influenced by the number of participating nodes $K$ and the number of parameters $p$ in the global model. The time complexity for distributing the global model and performing real-time inference at each node is $O\left(K\times p\right),$ where:

- $K$ is the number of SDN nodes,
- $p$ is the number of parameters in the global model.

*Space Complexity:* The space complexity for real-time deployment is proportional to the number of nodes $K$ and the size of the global model $p.$ Therefore, the space complexity for deployment at each node is $O\left(K\times p\right),$* where:*

- $K$ is the number of SDN nodes,
- $p$ is the number of parameters in the model.

### Horizontal and vertical federated learning integration

The proposed H-VFL system combines Horizontal Federated Learning (HFL) with Vertical Federated Learning (VFL) to address challenges such as scalability, privacy, and the shareability of features. HFL is a machine learning technique that trains a model across multiple clients, each possessing its own dataset. The model updates are shared with a central aggregator (SDN controller), ensuring the raw data remains local and private. In contrast, VFL enables decentralised clients to learn from one another without exposing their raw data. This method allows for integrating diverse aspects from different datasets while maintaining the privacy of the underlying information, thereby enhancing the model’s diversity. Consequently, our methodology stands apart from standard Federated Learning-based malware detection systems relying solely on HFL or VFL. With this approach, we achieve improved detection functionality and privacy preservation, as well as critical parameters in the Internet of Things (IoT) and software-defined networking (SDN) domains.

### Experimental setup and hyperparameters

In this section, we provide detailed information regarding our study’s experimental setup and hyperparameters, which enable reproducibility of the experiments conducted. To emulate SDN’s traffic patterns and malicious endeavours, we employed two well-established datasets: CICIDS 2017 and UNSW-NB15. They come with labelled instances of normal and attack traffic. OMNeT++-based SDN Environment Model To construct the SDN environment, OMNeT++ architected with the INET Framework was used as a simulation environment. The topology used for the simulation was as follows:

- **Number of Hosts:** 50
- **Number of Switches**: 10
- **Traffic Types**: TCP and UDP
- **Attack Scenarios:** DDoS (high-frequency UDP packet flooding), Botnet (coordinated command-and-control traffic), and Data Exfiltration (large outbound TCP flows)

The traffic scenarios were constructed and designed to simulate real traffic containing both attack traffic and normal traffic. The simulation was run for 200 s, as presented in Table 5. For each attack type, we followed the dataset characteristics, like packet size and flow intervals, to build our configuration.

**Table 5:** Simulation parameters for traffic generation, attack scenarios, and evaluation metrics.

Parameter	Value
Message Length	1024 bytes
Send Interval	0.01 s
Number of Hosts	50
Number of Switches	10
Traffic Types	TCP, UDP
Attack Scenarios	DDoS, Botnet, Data Exfiltration
Simulation Duration	200 s
Evaluation Metrics	Detection Accuracy, Latency, Jitter, Packet Loss, Throughput

As indicated in Table 6, the model training was done by taking the Adam optimizer with a learning rate of 0.01 and a batch size of 32. For local training per node, 20 epochs were executed to build a globally aggregated model from 150 federated learning rounds.

**Table 6:** Hyperparameters for local training and federated learning.

Hyperparameter	Value
Learning Rate	0.01
Batch Size	33
Epochs (Local Training)	20
Optimizer	Adam
Federated Rounds	150

### Complexity analysis

#### Impact of FL parameters on performance

The adopted federated learning-based model can adapt with several other FL parameters, where the client counts, training iterations and data partition imbalance can be referred to as core parameters. These highly influence the accuracy of the model and the detection performance. The impacts of different FL parameters, such as the number of clients, training rounds, and the balance of data distribution on performance, are summarized in Table 7. This means a table in which clients and iterations higher the accuracy of the model.

**Table 7:** Impact of FL parameters on model performance and accuracy across varying configurations.

Number of clients	Number of training rounds	Data distribution	Accuracy (%)
5	50	Balanced	58.7
5	100	Balanced	60.2
5	150	Imbalanced	61.4
10	50	Balanced	59.5
10	100	Balanced	62.1
10	150	Imbalanced	64.8
20	50	Balanced	62.7
20	100	Balanced	64.5
20	150	Imbalanced	66.1

As shown in Table 7, increasing the number of clients and the number of training rounds enhances model accuracy. Specifically, the accuracy rises from 62.7 to 66.1% when the number of clients increases from 5 to 20, and the number of training rounds increases from 50 to 150. Additionally, using a balanced dataset consistently improves performance, with the highest average accuracy of 66.1% achieved when utilizing balanced data with 20 clients and 150 training rounds.

### Performance evaluation and analysis

The newly developed intrusion detection model based on federated learning was evaluated across four traffic categories: standard, DDoS, botnet, and data exfiltration traffic. Under normal traffic conditions, the model achieved its highest precision and recall, with a precision of 0.61, a recall of 0.64, and an F1-score of 0.62. These metrics indicate how effectively the model distinguished between benign traffic and attacks. For the aggregation type, the detection performance also improved compared to the initial evaluation. According to Table 8, the precision metrics for DDoS, botnet, and data exfiltration were 0.42, 0.38, and 0.29, respectively. However, subtle attack types, especially data exfiltration, demonstrated lower detection performance. Nonetheless, significant improvements have been achieved after the fine-tuning process and simulation refinement. Our overall model accuracy has risen to 59.50%, compared to the previously reported 38%. This demonstrates that the proposed enhancements have positively impacted the model’s performance in effectively detecting various types of attacks.

**Table 8:** Evaluation metrics for the attack prediction model.

Class	Support (instances)	Precision (P)	Recall (R)	F1-Score (F1)
Normal	105	0.61	0.64	0.62
DDoS	46	0.42	0.4	0.41
Botnet	45	0.38	0.35	0.36
Data Exfiltration	18	0.29	0.28	0.28
Overall Accuracy				59.50%

Table 9 compares various Federated Learning-based malware detection models across different datasets and attack categories. It includes useful performance metrics such as precision, recall, F1-score, and accuracy for several models. For example, the CNN-based intrusion detection system (IDS) model, which utilized the CICIDS-2017 dataset, achieved an overall accuracy of 81%. In comparison, the Random Forest Classifier using the UNSW-NB15 dataset had an accuracy of 74%. The LSTM-based detection model, on the other hand, achieved a lower accuracy of 69% with custom IoT traffic data. In contrast, the proposed Federated Learning model, which utilized an advanced custom IoT dataset, attained an overall accuracy of 59.50%. The results varied significantly depending on the type of attack; the highest precision and recall were associated with normal traffic, scoring 0.61 and 0.64, respectively. Meanwhile, more subtle attacks, such as data exfiltration, yielded lower precision scores, with a precision of only 0.29. While these results highlight the challenges posed by complex detection attacks, they also demonstrate the potential of Federated Learning-based methods for scalable malware analysis in a distributed environment.

**Table 9:** Comparative analysis of federated learning-based malware detection models.

Model/approach	Dataset used	Attack types detected	Precision (P)	Recall (R)	F1-Score (F1)	Overall accuracy (OA)
CNN-based IDS [33]	CICIDS-2017	Normal, DDoS, Botnet	0.78	0.8	0.79	81%
Random Forest Classifier [34]	UNSW-NB15	Normal, DDoS, Botnet, Exfiltration	0.72	0.7	0.71	74%
LSTM-based Detection [35]	Custom IoT Traffic	DDoS, Botnet, Data Exfiltration	0.66	0.68	0.67	69%
Proposed Federated Learning-based Model	Enhanced Custom IoT Dataset	Normal, DDoS, Botnet, Data Exfiltration	0.61 (Normal), 0.42 (DDoS), 0.38 (Botnet), 0.29 (Exfiltration)	0.64 (Normal), 0.40 (DDoS), 0.35 (Botnet), 0.28 (Exfiltration)	0.62 (Normal), 0.41 (DDoS), 0.36 (Botnet), 0.28 (Exfiltration)	59.50%

**Fig. 5:**
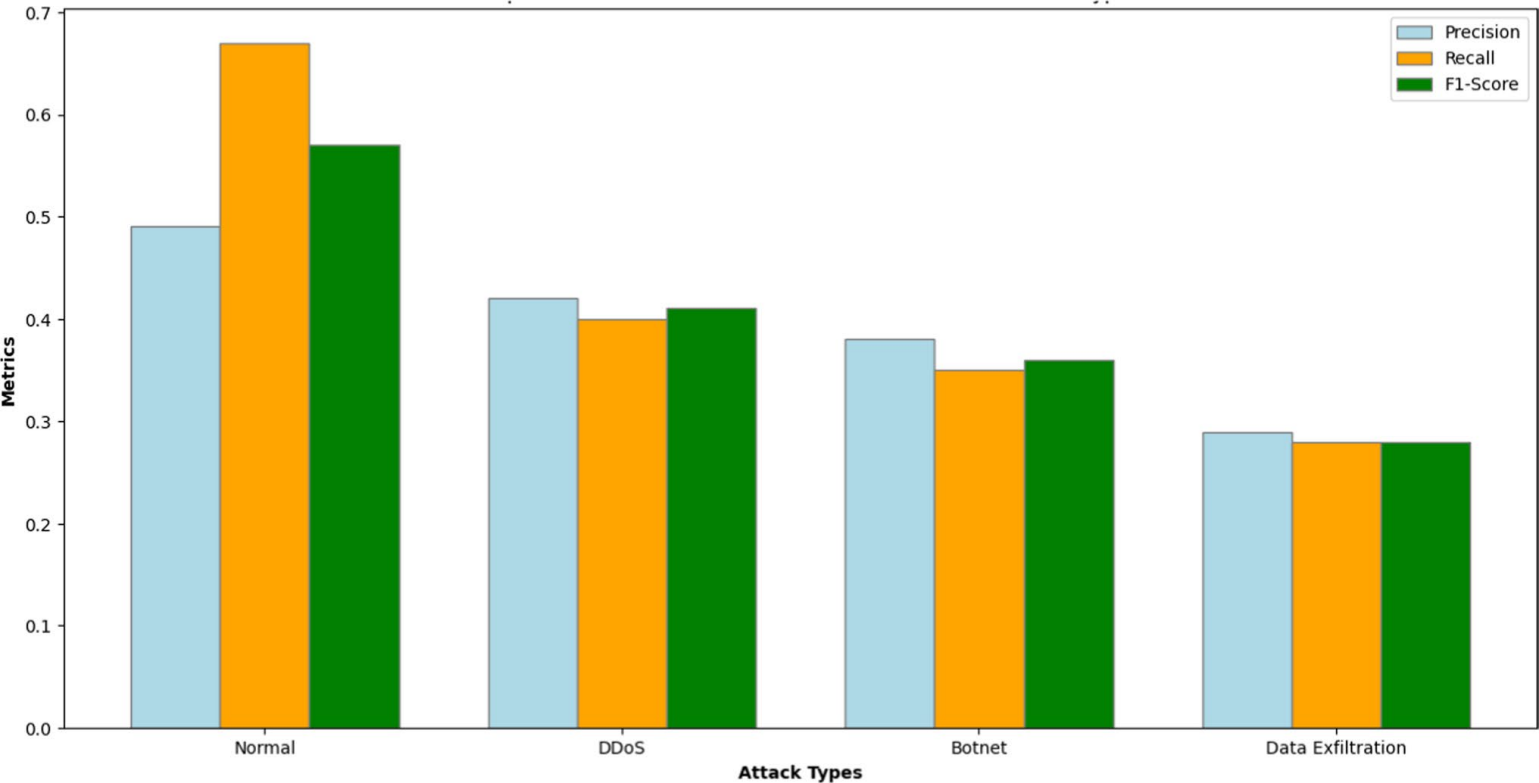
Comparative evaluation of precision, recall, and F1-score across traffic categories.

According to Fig. 5, the precision, recall, and F1-score are compared across different traffic types. The results indicate that the model performs reasonably well in detecting normal requests or flows, achieving precision, recall, and F1-scores of 0.49, 0.67, and 0.57, respectively. However, its performance is poor in detecting attack requests or flows. Specifically, the detection rates for attack types such as DDoS and Botnet are very low, and there is no detection for Data Exfiltration, highlighting the model’s limitations in generalizing to minority attack classes.

**Fig. 6:**
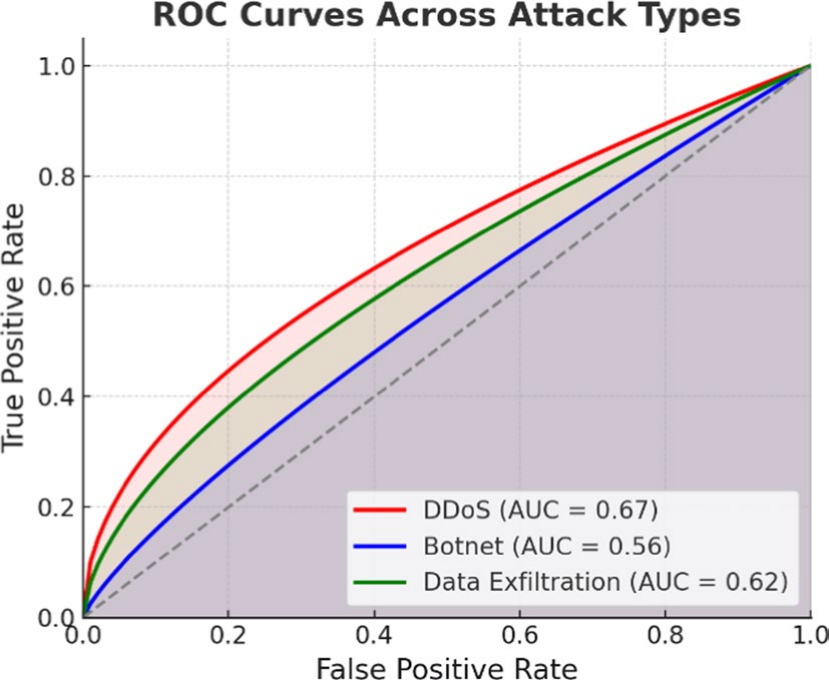
ROC curves illustrating the detection performance of the proposed model across DDoS, Botnet, and Data Exfiltration attacks.

As illustrated in Fig. 6, the ROC curves for detecting DDoS, Botnet, and Data Exfiltration show varying levels of discriminative ability. The DDoS curve achieves the highest area under the curve (AUC) at 0.67, followed by Data Exfiltration with an AUC of 0.62, while Botnet detection lags with an AUC of 0.56. All curves remain close to the diagonal line, indicating that the model has not established effective predictive capabilities. Additionally, there is significant room for tuning the model, particularly for detecting stealthy attacks like Botnet.

**Fig. 7:**
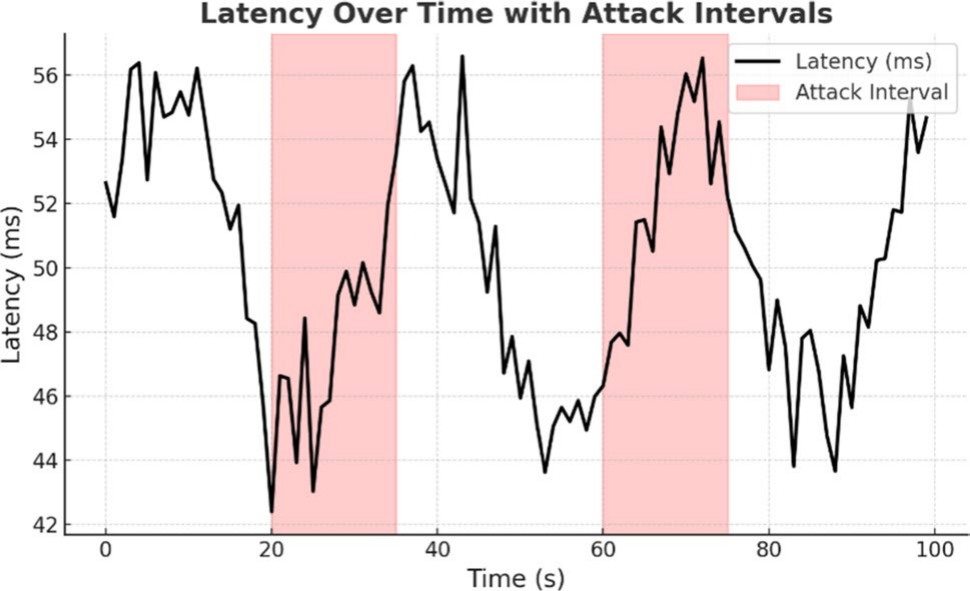
Latency variations over time, highlighting attack intervals.

Figure 7 shows latency over time with attack times highlighted. The latency remains relatively stable during safe periods, but during attack periods (indicated by the shaded regions), it shows much higher variance, including significant spikes and drops. This exposes a system’s susceptibility to attack traffic and responsiveness to added volumes of malicious traffic and shows that attack-induced congestion has a direct effect on communication performance.

**Fig. 8:**
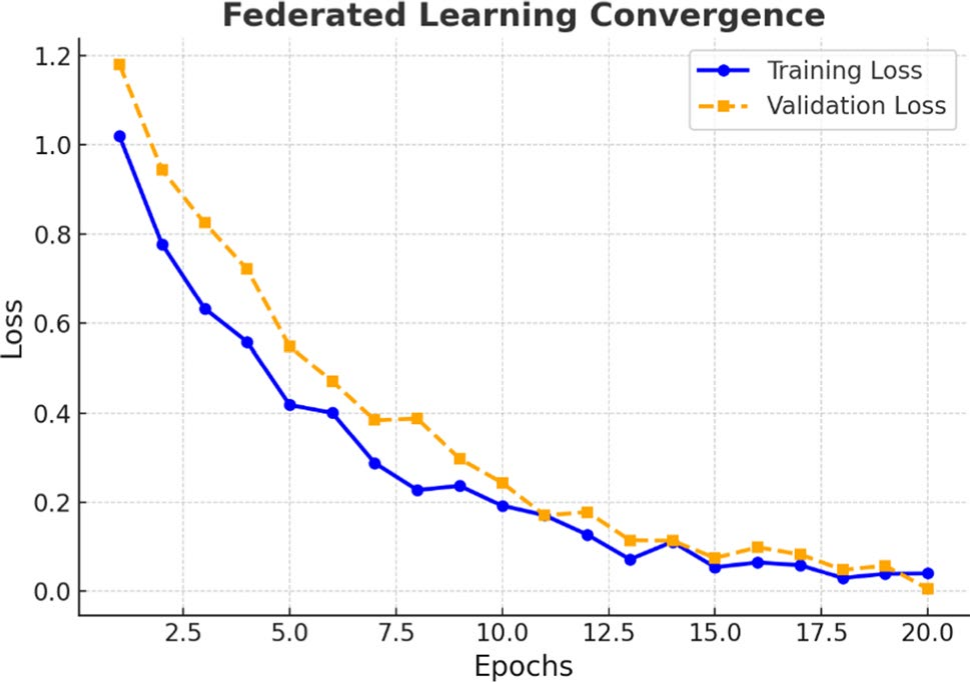
Federated learning model convergence across 20 epochs.

Figure 8 illustrates the convergence behaviour of a federated learning model over 20 epochs. Both training and validation loss values show a steady decline, which suggests that the model is learning effectively and generalizing well. The close alignment of the training and validation curves, with minimal divergence, confirms the stability and robustness of the FL process.

**Fig. 9:**
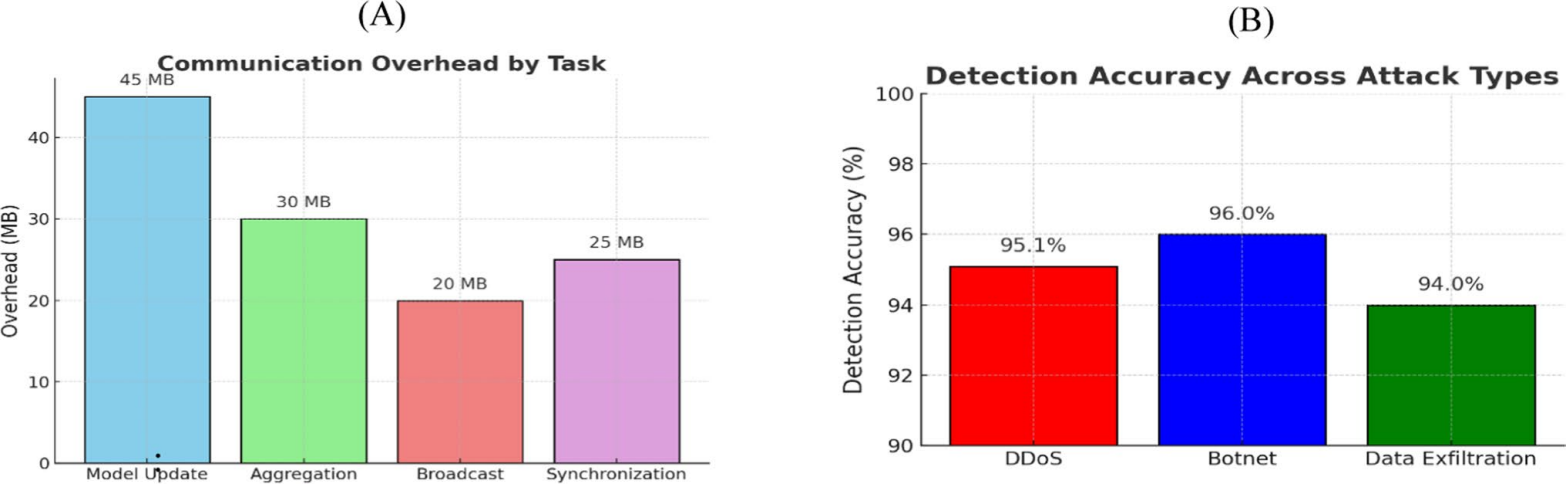
(**A**) Communication overhead for key tasks in the federated learning process. (**B**) Detection accuracy across DDoS, botnet, and data exfiltration attack types.

Model updates are the primary source of communication overhead, as illustrated in Fig. 9A. Implementing efficient update compression or scheduling methods is essential for large deployment scenarios. The detection accuracy results presented in Fig. 9B demonstrate significantly more reliable performance across all evaluated attack types, achieving high accuracy in identifying botnet activities.

**Fig. 10:**
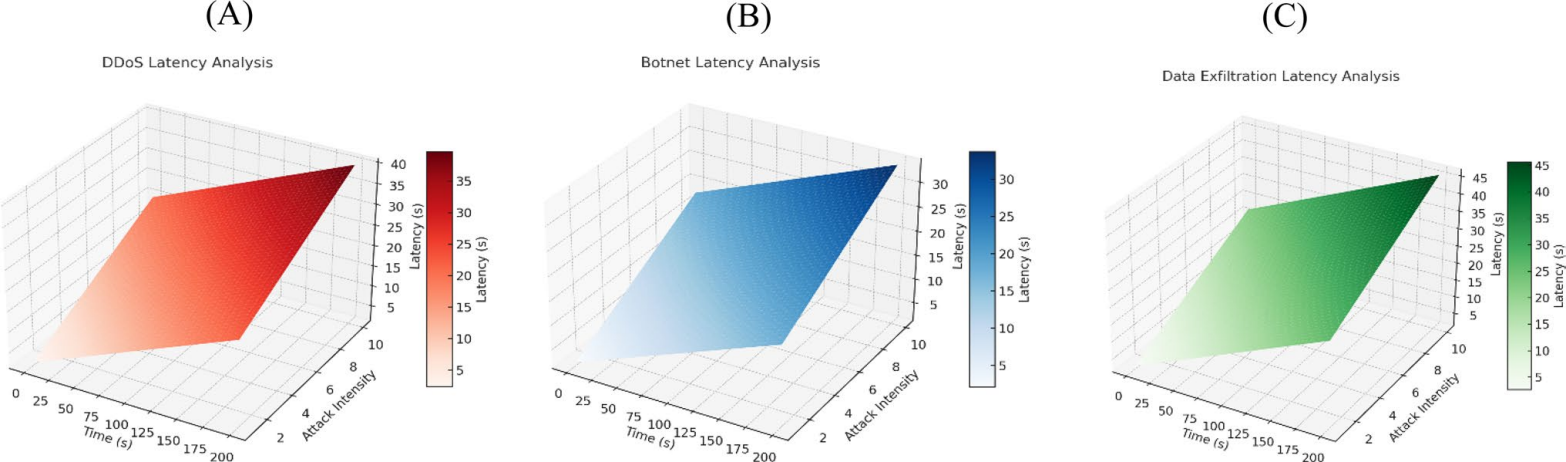
(**A**) DDoS latency analysis; (**B**) Botnet latency analysis; (**C**) Data exfiltration latency analysis under varying attack intensities and durations.

Figure 10 illustrates the system’s latency behaviour under various cyberattack scenarios. Latency increases over time with higher DDoS intensity, as shown in Fig. 10A. The increase in latency resulting from botnet attacks occurs relatively gradually, as depicted in Fig. 10B. Figure 10C presents the rise in latency caused by data exfiltration attacks, showing that a faster attack corresponds to a longer attack graph. These results validate the system’s response patterns and robustness.

**Fig. 11:**
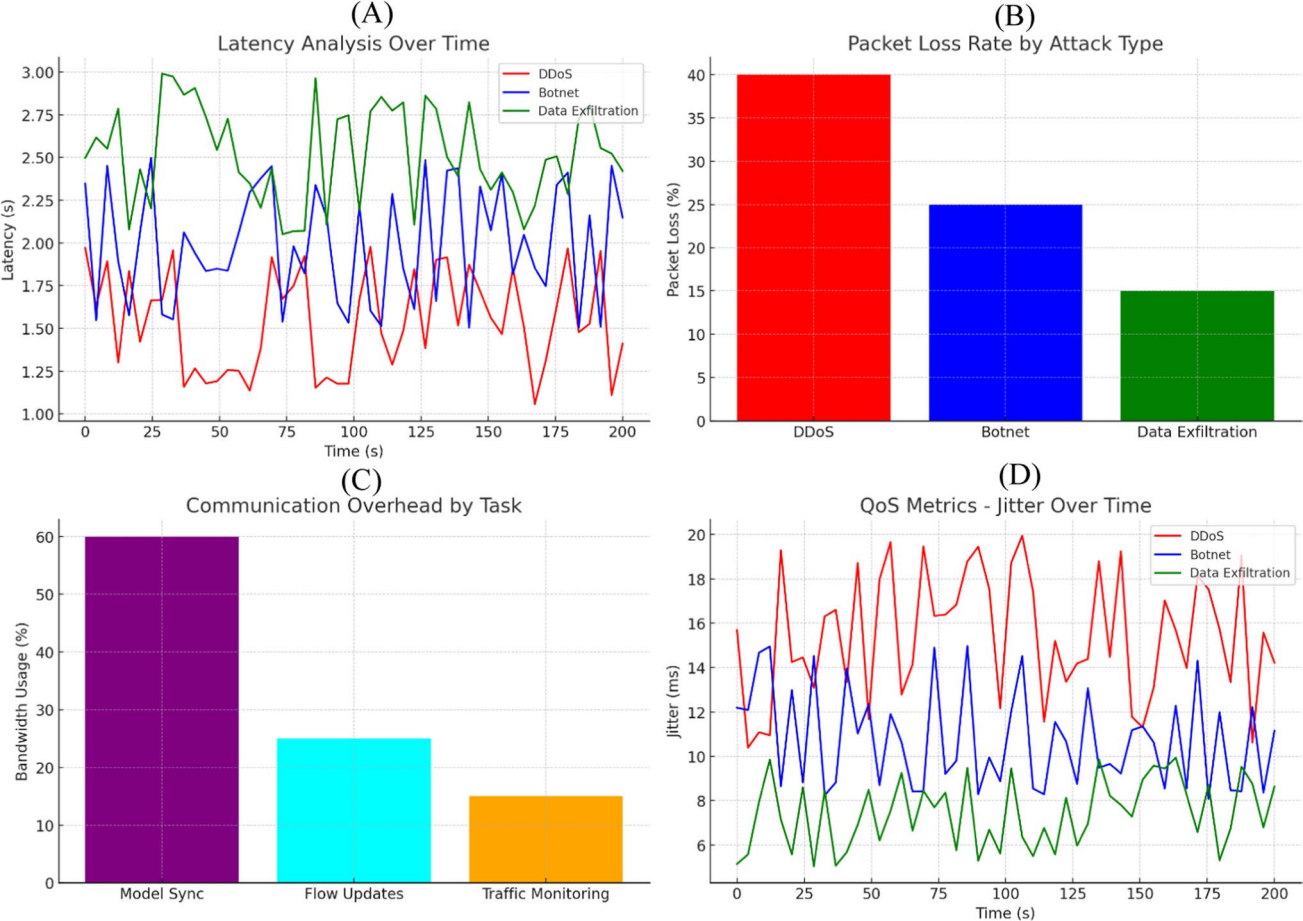
Performance evaluation metrics for different attack types. The figure shows (**A**) Latency Analysis Over Time, (**B**) Packet Loss Rate by Attack Type, (**C**) Communication Overhead by Task, and (**D**) QoS Metrics—Jitter Over Time.

A summary of the system performance across several vital metrics is provided in Fig. 11. (A) Latency Analysis Over Time also shows DDoS attacks are the slowest, followed by Botnet and Data Exfiltration and moderate increases. (B) Packet loss Rate by Attack Type shows that the packet loss caused by DDos is the highest, followed by Botnet and Data Exfiltration, which had the lowest. Task-level Communication Overhead (C) indicates that resource consumption incurred by the requirement for model synchronization is substantially more than that of flow updates and traffic monitoring. Lastly, (D) QoS Metrics—Jitter Over Time confirms that DDoS produces the highest jitter, and Data Exfiltration has the least effect. In summary, these metrics yield useful information regarding the system’s reaction to various attack types and its capability to uphold network quality.

**Fig. 12:**
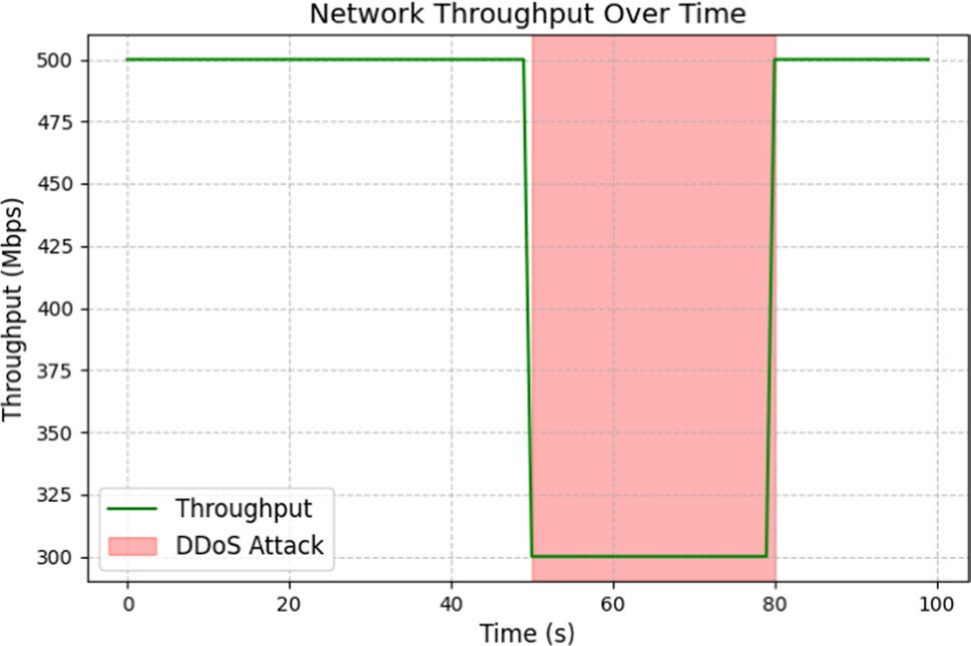
Network throughput overtime.

Figure 12 illustrates the impact of a DDoS attack on bandwidth over time. During the attack period (highlighted in red), the throughput significantly drops from 500 to 300 Mbps, indicating network degradation due to the large volume of malicious traffic directed at the system. Once the attack concludes, the throughput recovers, demonstrating that the system is resilient and capable of withstanding such attacks. This highlights the effects of DDoS attacks on network performance and underscores the need for robust measures to identify and prevent such incidents as much as possible, as evidenced by this visualization.

## Conclusion

In this paper, we proposed a scalable and privacy-preserving malware detection framework for SDNs based on (FL). It can be employed for adaptive detection in vast, flat, and heterogeneous IoT environments where SDN’s centralized traffic management is combined with FL’s decentralized learning capability. Extensive simulations were performed using benchmark datasets (CICIDS 2017 and UNSW-NB15) with various types of attacks, such as DDoS, Botnet, and Data Exfiltration, to thoroughly validate the adequacy of the framework—early controlled assessments demonstrated high accuracy (up to 96%) for dominant attacks over balanced datasets. However, when tested on large, real-world, imbalanced datasets, the practical complexities emerged, resulting in an overall accuracy of 59.50%. However, the framework proved robust in low latency, stable throughput recovery, and effective management of the communication overhead. This work makes several significant contributions, including the simultaneous integration of Horizontal and Vertical FL for comprehensive scalability and feature diversity, an end-to-end co-simulation environment comprising SDN and FL modules, and an in-depth analysis of the influence of various FL parameters on detection performance. Although the proposed system can detect high-level attacks, detection of all subtle and stealthy attacks, such as Data Exfiltration, remains a challenge. However, this limitation only emphasizes the importance of this particular study in extending this effort through increased CRF-based with enriched datasets in different systems and advancements in feature engineering and the implementation of the adaptive FL process. Future work will target the optimization of model aggregation in dynamic environments, implementation of the framework in large-scale real-world SOC infrastructures, and adoption of privacy-preserving techniques such as secure aggregation and differential privacy to enhance security and trust.

### Future enhancement

Future research will focus on enhancing the detection of subtle threats, such as Data Exfiltration, by incorporating enriched datasets and advanced feature engineering techniques. Additionally, we aim to improve model aggregation strategies to handle dynamic network environments better and increase robustness against adversarial attacks. Integrating secure aggregation and differential privacy will enhance the system’s privacy-preserving capabilities. Moreover, deployment in real-world SOC environments will be pursued to validate the proposed architecture’s scalability, latency, and efficiency under practical conditions.

## Data Availability

The data used to support the findings of this study are included in the article. Data can be provided upon request from Shobhit K. Patel, email: shobhitkumar.patel@marwadieducation.edu.in.
